# Evaluation of the External Rotation of the Femur Component in Functionally Aligned Robotic-Assisted Total Knee Arthroplasty

**DOI:** 10.7759/cureus.62948

**Published:** 2024-06-23

**Authors:** Sanjay B Londhe, Ravi Teja Rudraraju, Ravi Vinod Shah, Govindkumar Baranwal, Suneet Velankar, Zara Namjoshi

**Affiliations:** 1 Department of Orthopedics, Criticare Asia Hospital, Mumbai, IND; 2 Department of Orthopedics, Apollo Hospitals, Hyderabad, IND; 3 Department of Orthopedics, Sri Venkata Sai (SVS) Medical College, Mahbubnagar, IND; 4 Department of Statistics, Criticare Asia Hospital, Mumbai, IND

**Keywords:** kinematic alignment, functional alignment, posterior condylar axis, transepicondylar axis, robotic assisted total knee arthroplasty

## Abstract

Background

The conventional total knee arthroplasty (TKA) for grade 4 knee arthritis lacks individualized strategies for determining femur component rotation, contributing to suboptimal clinical outcomes and heightened patient dissatisfaction.

Methods

One hundred consecutive active robotic-assisted TKA (RA-TKA) patients were retrospectively evaluated. The control group is the patients undergoing conventional TKA for grade 4 arthritis of the knee joint, where the femoral component is placed in a fixed 3-degree external rotation. The study aimed to explore the relationships between the posterior femoral axis of the functionally aligned TKA (FAA), the trans-epicondylar axis (TEA), and the posterior condylar axis (PCA). Specifically, it investigated whether there is a statistically significant difference in femoral component rotation between the functionally aligned TKA (FTKA) and the conventional 3-degrees of external rotation of the femoral component used in traditional TKA (C-TKA). Internal rotation is indicated by a negative value for the femur component. A student’s t-test was employed to compare mean rotation values between FTKA and C-TKA, with a p-value below 0.05 considered statistically significant.

Results

A total of 100 patients (male: female, 11:89) were studied. The FAA was externally rotated in relation to the TEA (mean 1.451°, SD 1.023°, p-value <0.0001). As regards the PCA, the FAA was externally rotated (mean 2.36°, SD 2.221°, p-value 0.0002). These findings demonstrate a statistically significant difference in femoral component rotation between FTKA and C-TKA. Clinically, no patellofemoral complications or premature loosening were observed at one-year follow-up.

Conclusion

Functional alignment TKA technique resulted in external rotation of the femur component with respect to TEA and PCA. This negates the null hypothesis, indicating a statistically significant difference amongst the femur component rotation implanted according to the FTKA concept with robotic assisted technology and C-TKA.

## Introduction

The rotation of the femoral component plays a crucial role in achieving favorable results following total knee arthroplasty (TKA). The internal or external rotation of the femur component in TKA significantly influences sustained clinical outcomes [1]. Malrotation or improper alignment of the femur component can potentially lead to complications such as patellofemoral maltracking, instability [2], and post-TKA stiffness [3]. It can also lead to anterior knee pain and instability during mid-flexion [4,5]. Malrotated femur components can lead to abnormal torsional loading on the tibia component [6], and impingement of the cam post in posterior stabilized (PS) cruciate sacrificing designs, causing premature wear and loosening of the components [7]. There is no universal agreement on determining femur component rotation during the TKA operation. The two different techniques used are measured resection and gap balancing [8,9]. In measured resection, generally used bony landmarks are trans-epicondylar (TEA) and posterior condylar (PCA) axes.

Studies on cadavers and radiography demonstrate that the TEA is parallel to the flexion-extension axis of the knee [6,7]. The intraoperative measurement of the TEA involves connecting the most prominent point of the lateral epicondyle with the center of the medial epicondyle sulcus. The knee joint's flexion/extension axis may be accurately replicated, and the rectangular gap achieved by using this landmark has been proven to be highly reliable [10,11]. The visualization of the TEA during the procedure is challenging as the bony landmarks used for determining the TEA are covered with soft tissues (especially the medial epicondyle sulcus) [8]. In view of this difficulty, PCA has been utilized as a surrogate for TEA during the TKA procedure. Various studies have tried to evaluate the usefulness of PCA and TEA for accurately placing the femur component in proper rotation during TKA [4,7,12,13,14,15]. In conventional TKA, usually, the femur zigs set the femur component in 3-degrees of external rotation with respect to PCA. However, multiple studies have shown that this method using PCA as a surrogate for the TEA may cause femur component malrotation in 25.5% to 72% of TKA [12,13,14,15].

As technology has advanced, the concept of functional alignment in TKA (FTKA) offers a step up from kinematic alignment. Using robotic-assisted technology (RA-TKA) to refine bone resection, implant location, and/or soft tissue releases, FTKA improves TKA function according to each patient's unique alignment, bone structure, and soft tissue features surrounding the knee [16]. Functional alignment (FA) is designed to restore the joint's native plane and obliquity as determined by the soft-tissue envelope. This technique involves preoperative planning to achieve natural mechanical alignment (NMA), positioning components perpendicularly to the axes of the femur and tibia [3]. Surgical aids like computer navigation or robotic technology assess resection thickness, flexion-extension gaps, and limb alignment during surgery. Osteophytes are removed, and varus or valgus strains are applied to restore native periarticular soft-tissue tension and correct coronal plane deformities. Computer software virtually adjusts component positions, displaying potential changes in limb alignment, flexion-extension gaps, and range of motion (ROM). Preserving the joint line height enhances knee flexion, aids patella tracking, and improves mid-flexion stability [3]. This technique aims for individualized physiological limb alignment within the 0° to 3° safe zone of coronal alignment and patient-specific knee kinematics while minimizing soft-tissue releases. Ligamentous releases may be necessary for fixed deformities to balance flexion-extension gaps but are less frequent compared to standard NMA techniques. Advances in technology have refined FTKA, particularly with RA-TKA, improving function based on each patient's unique alignment, bone structure, and soft tissue features [3]. Preoperative imaging creates detailed 3D knee models, enabling precise implant position planning in the sagittal, coronal, and axial planes. FA targets a hip-knee-ankle (HKA) angle of 0° with an acceptable deviation of ±3° (177°-183°), replicating the patient’s natural alignment. Accurate bone resection and implant positioning ensure that the femoral implant fits precisely, without an overhang or notching [16]. Implant rotation aligns closely with the trans-epicondylar axis (TEA) for natural patellofemoral kinematics. Robotic-assisted systems, such as the fully automated Cuvis Joint robot (Curexo Inc., Korea, supported by Meril Healthcare Pvt. Ltd., India), enhance precision and reproducibility. Evaluating the PCA and posterior femoral axis determines external rotation relative to the TEA, matching the patient's natural anatomy. FTKA results in improved joint function, personalized treatment, reduced complications, and enhanced implant longevity, marking a significant advancement in personalized knee replacement surgery. The study aims to explore the relationships between the posterior femoral axis of the functionally aligned TKA (FAA), TEA, and PCA in patients undergoing RA-TKA. The data from this study was presented at the World Arthroplasty Congress, jointly hosted by the European Knee Society, European Hip Society, Knee Society USA, and Hip Society USA, in Madrid, Spain, on April 18-19, 2024.

This article was previously posted to the Research Square preprint server on April 11, 2024. However, the article is not under consideration in any other journal.

## Materials and methods

**Figure 1:**
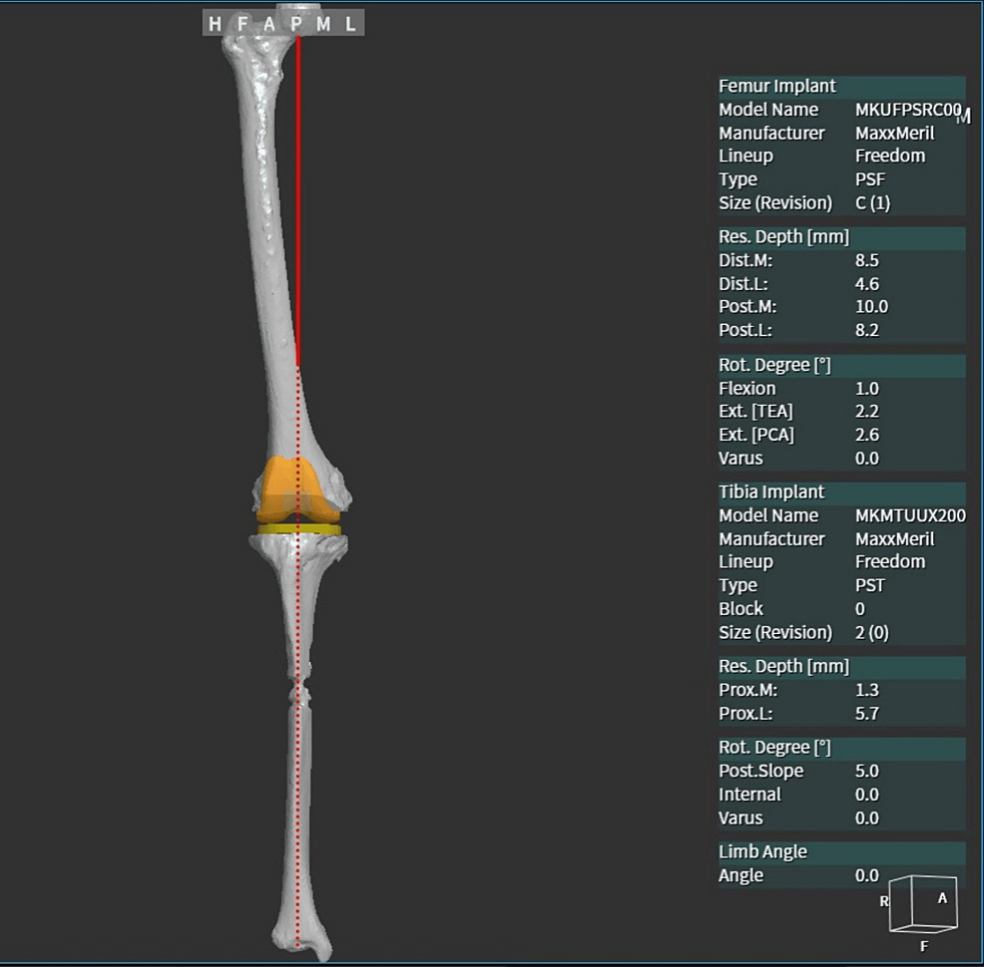
A representative image of the preoperative planning based on the preoperative 3D CT scan.

The study was conducted at Criticare Asia Hospital, Mumbai, India. We present a single-center, retrospective assessment of data gathered from prospectively enrolled patients operated on by a team of orthopedic surgeons led by a senior surgeon who underwent RA-TKA with the fully autonomous Cuvis Joint robot. Due to the study's retrospective nature, the local research ethics committee waived approval. An independent observer retrospectively reviewed 100 consecutive patients who underwent active RA-TKA. The study comprised patients with grade 4 arthritis of the knee joint receiving RA-TKA. Individuals who previously had knee surgery were excluded from the study. One week prior to the procedure, patients were required to have a preoperative CT scan of the leg scanned in hip, knee, and ankle (HKA) regions. The scanned images were transferred in the .jpg format. By using a proprietary operating system and a dedicated laptop, segmentation and preoperative planning were performed. The planned alignment of the limb was an HKA angle of 0 degrees with an acceptable deviation of -3 to +3 degrees (177-183 degrees) during the execution of the functional alignment while performing the TKA procedure (Figure 1).

**Figure 2:**
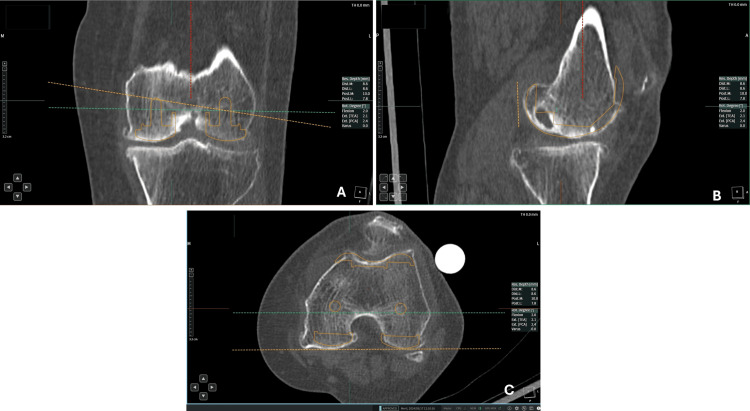
A representative image of a femoral implant fit in the coronal (A), sagittal (B), and axial (C) planes.

The planning process involved establishing the HKA center, selecting the tibia and femur bony landmarks, obtaining precise implant positions in the sagittal, coronal, and axial planes, and determining the bony resection measurements at distal, anteroposterior (AP), anterior and posterior chamfer cuts, and proximal cuts of the tibia. The femur implant that fit precisely in all three planes without overhanging or notching was chosen (Figure 2).

**Figure 3:**
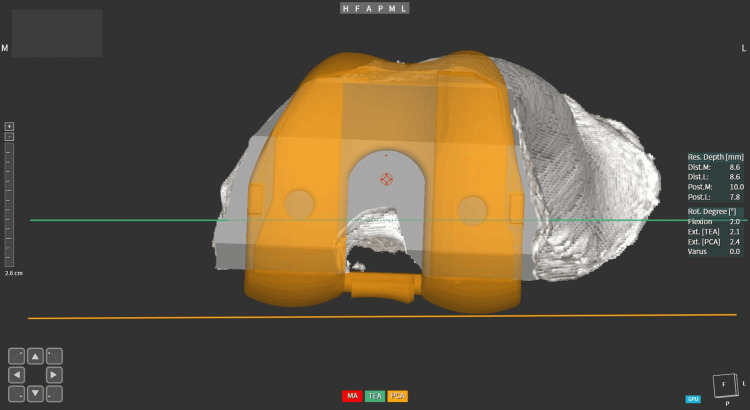
The trans-epicondylar (TEA), posterior condylar (PCA) axes, and posterior femoral axis (FAA) of the functionally aligned TKA (FTKA). TKA: total knee arthroplasty

The implant rotation was kept as close as possible to TEA, and the negative value denoted the relative internal rotation of the femur component. The TEA, PCA, and posterior femoral axis (FAA) were evaluated on the preoperative 3D CT scan conducted by an independent observer who was not a member of the core surgical team. The correlation between FAA, TEA, and PCA was determined (Figure 3). The control group was patients with conventional TKA, where a standard 3-degrees of external rotation is given to the femoral implant with the help of traditional jigs.

The study's null hypothesis was that there would not be a statistically significant difference between FAA and the standard 3-degrees of external rotation applied during conventional total knee arthroplasty (C-TKA). The statistical analysis was carried out with the student's t-test. The mean rotation values were compared between FTKA and CTKA. A P-value of < 0.05 was considered significant.

## Results

We conducted a study involving 100 consecutive patients diagnosed with grade 4 arthritis of the knee joint. Patients’ distribution was 89% female and 11% male, and the average age of the participants was 68.3 years (Table 1).

**Table 1:** Patient demographics undergoing robotic assisted total knee arthroplasty.

Demographics	n=100
Mean age, years	68.3
Body mass index, Kg/m^2^	27.4±5.2
Males, n	11
Females, n	89
Left knees, n	48
Right knees, n	52

Preoperative deformities were categorized as varus and valgus deformities. Ninety-four out of 100 patients had varus alignment (range 7 to 18 degrees), and six patients had valgus alignment (range 6 to 14 degrees). Table 2 shows the details of the preoperative deformity.

**Table 2:** Specifics regarding the preoperative deformity in the study.

Procedural characteristics	
Total no of subjects	100
Varus knees, n (range)	94 (7° to 18°)
Valgus knees, n (range)	6 (6° to 14°)

A broad range of variability was observed among the FAA, TEA, and PCA. The FAA in correlation to TEA was consistently observed to be externally rotated, with a mean external rotation of 1.451°±1.023°. This implies a systematic and statistically significant external rotation, as evidenced by the p-value of <0.0001. The tight range further underscores the consistency of this observed rotation among the study population.

Similarly, the analysis of the PCA also demonstrated an external rotation of the FAA. The mean external rotation measured 2.36° with a standard deviation of 2.221°. The associated p-value of 0.0002 reinforces the statistical significance of this finding. Table 3 shows the relationship of FAA to TEA and PCA. The consistency in external rotation observed across both TEA and PCA comparisons implies a robust and reliable pattern in the functional alignment of the TKA. These results contradict the null hypothesis, demonstrating a statistically significant difference in femoral component rotation when implemented according to the FTKA concept with robotic-assisted technology as opposed to C-TKA. Clinically, none of the 100 patients experienced any patellofemoral complications or premature loosening at the one-year follow-up.

**Table 3:** Femoral external rotation concerning transepicondylar axis (TEA) and posterior condylar axis (PCA) in robotic-assisted total knee arthroplasty. TEA: transepicondylar axis; PCA: posterior condylar axis; n: number of patients; SD: standard deviation

n=100	Femoral rotation with TEA (degrees)	Femoral rotation with PCA (degrees)
Mean value (SD)	1.451 (±1.023)	2.36 (±2.221)
P-value	<0.0001	0.0002

## Discussion

The primary outcome of our research indicates that the FAA is externally rotated by 1.45 and 2.36 degrees in relation to the TEA and PCA, respectively. Additionally, the study reveals a difference of 0.91 degrees between TEA and PCA. Consequently, the null hypothesis is negated, signifying a statistically significant variance observed among the FAA and standard 3-degrees of external rotation utilized in C-TKA. Precise alignment and rotation of implants play a pivotal role in the success of TKA, as any misalignment can result in issues like abnormal wear, premature loosening, and patellofemoral issues [16,17,18]. The fusion of navigation and robotic surgery has ushered in the functional alignment concept, enabling surgeons to utilize 3D simulations and real-time ligament tension data for precise adjustments during knee arthroplasty. Research indicates that RA-TKA technology excels in gap balancing and accurately restores the joint line while preserving near-normal knee kinematics [19]. The improved precision of component placement in the sagittal plane with RA-TKA potentially facilitates greater accuracy in knee gap balancing than C-TKA [20]. The authors of a previously published prospective randomized study compared the patients who underwent RA-TKA and C-TKA. They noted an early increase in maximum knee flexion in the RA-TKA cohort (104.1°, 90°-120°) compared to the C-TKA (93.3°, 90°-110°) (p-value <0.001), along with a noticeable and gradual reduction in post-operative stiffness in the RA-TKA cohort [21]. The reported resections of the femoral as well as tibial components (coronal plane) in RA-TKA cases were oriented within ±3° and the accuracy of implant alignment in the RA-TKA cohort was 94.7% for the femoral component and 95% for the tibial components, whereas in C-TKA cases, the percentages were 87.2% for the femoral components and 82.1% for the tibial components [22].

Functional alignment’s primary goal is to position TKA components to reconstruct the joint's plane and obliquity while minimizing damage to soft tissues. Unlike kinematic alignment, functional alignment not only considers bony anatomy but also ensures a balanced alignment of soft tissues [23]. For satisfactory and optimal TKA outcomes, ensuring accurate rotational alignment of the femoral component is important. While excessive external rotation may cause post-operative pain (including pain in the anterior part of the knee), flexion and mid-flexion instability, and stiffness, internal rotation results in patellar maltracking and instability [20,24]. In a study assessing 190 CT scans, it was observed that the native femur was in 3° internal rotation in 65% of the healthy knee joints [25]. The progression of osteoarthritis may increase this rotation because of elevated soft tissue stress caused by growing malalignment of the lower limb. To ensure uniform flexion-gap alignment during TKA, the femoral component should be rotated in the opposite direction with the equivalent degree value [24]. Optimal functionality frequently correlates with an external rotation of the femoral component ranging from 3° to a maximum of 5° relative to the posterior condylar line or positioning it at 0° in relation to the transepicondylar line [18]. The two common landmarks used while deciding the femur component rotation in conventional TKA are TEA and PCA. Several authors suggest utilizing the epicondylar axis as a point of reference for bone cutting and ligament balances to create the most favorable flexion-extension axis [26]. A surgeon’s skill and experience with TKA play a crucial role in achieving femoral rotation alignment through the TEA during the TKA procedure. Prior studies have reported approximately 86.5% of knees to be within a 5° range of the true epicondylar axis [27], while one study noted 90% of them deviated by less than 3° using the TEA [5], and another study reported rotation in 90.8% of cases under 5° from the surgical transepicondylar axis [28].

Techniques that use the PCA as a reference assume that it represents the neutral axis of femoral rotation. In the research conducted by Benjamin et al., it was shown that the PCA predominantly matched the rotational positioning of the implanted femoral component in 62% of cases, falling within ±1° [29]. Due to individuals’s varying wear patterns of the posterior condylar cartilage, it is not appropriate to generalize a fixed alignment of 3° of external rotation relative to the posterior aspect of the femoral condyles. Employing a 3° external rotation factor based on such assumptions may result in excessive external rotation, resulting in unfavorable clinical outcomes [30]. Our study shows that femoral external rotation with respect to PCA is 2.36±2.221 degrees (as opposed to a fixed angle of 3-degrees), indicating statistical significance with a p-value <0.0002.

The results of our study, involving femoral rotation with respect to TEA and PCA, demonstrated consistent external rotation in the functional alignment. The mean values, standard deviations, and p-values provided valuable insights into the biomechanics of osteoarthritis and the relationships between TEA, PCA, and FAA. Our findings suggest that using robotic preoperative software to identify surgical TEA is a more reliable technique than a fixed 3° external rotation to PCA to achieve adequate femoral component rotation during TKA. In light of this data, we recommend caution when using a fixed PCA to set femoral component rotation during TKA, as it may result in a high incidence of outliers.

Limitations

The first limitation is that our planning for setting the femur component rotation does not account for the gap balancing philosophy method. The gap balancing method initially achieves a balanced gap in extension. Subsequently, it adjusts the rotation of the femur component to attain a balanced rectangular gap relative to the upper tibia resection. The advantage of the RA-TKA is that it mainly depends on a measured resection philosophy, thereby reducing the necessity of soft tissue releases. The second limitation is that the study presents a small sample of patients of one ethnicity. Further studies are required at multiple centers involving a greater number of patients of different ethnicities. The third limitation is that the study did not evaluate patient-reported outcomes (PROMS). Further extension of the present study is ongoing, comparing the PROMS of this cohort versus the C-TKA cohorts. To the best of our knowledge, the study's strength lies in its novelty, in which we assessed the relationship between the posterior femoral axis of functionally aligned TKA and commonly used axes (i.e., TEA and PCA) for determining femur component rotation in the conventional method of performing TKA. Our study demonstrated that the FAA significantly differs from the routine 3-degrees of external rotation imparted in the C-TKA using currently available alignment instruments.

## Conclusions

In conclusion, our investigation into the functional alignment concept in total knee arthroplasty has yielded compelling results in a cohort of one hundred consecutive patients. The consistent external rotation observed in the functional alignment axis in relation to both the TEA and the PCA underscores a distinct departure from traditional alignment approaches. This aligns seamlessly with the fundamental principles of FTKA.

The statistical significance of the observed external rotation emphasizes the need for a paradigm shift towards more patient-specific treatments. Our findings challenge the conventional practice of applying a uniform femur component external rotation, often around 3-degrees, across all patients. Instead, we advocate a more individualized and tailored approach, accounting for the unique characteristics of each patient's knee, which is in alignment with the principles of the functional alignment concept in total knee arthroplasty.
